# Inhibition of BMP3 increases the inflammatory response of fibroblast-like synoviocytes in rheumatoid arthritis

**DOI:** 10.18632/aging.103422

**Published:** 2020-06-22

**Authors:** Biao Song, Xiaofeng Li, Qingqing Xu, Suqin Yin, Sha Wu, Xiaoming Meng, Cheng Huang, Jun Li

**Affiliations:** 1 Anhui Province Key Laboratory of Major Autoimmune Diseases, Anhui Institute of Innovative Drugs, School of Pharmacy, Anhui Medical University, Hefei 230032, China; 2 The Key Laboratory of Anti-inflammatory and Immune Medicines, Ministry of Education, Hefei 230032, China

**Keywords:** adjuvant-induced arthritis, BMP3, inflammatory cytokines, chemokines

## Abstract

Rheumatoid arthritis (RA) is a persistent autoimmune disease. Fibroblast-like synoviocytes (FLS) are a key component of invasive pannus and a pathogenetic mechanism in RA. Expression of bone morphogenetic protein 3 (BMP3) mRNA is reportedly decreased in the arthritic synovium. We previously showed that BMP3 expression is significantly downregulated in the synovial tissues of RA patients and models of adjuvant-induced arthritis (AIA). In the present study, we explored the association between BMP3 and FLS migration and secretion of proinflammatory factors in RA. We found that inhibition of BMP3 expression using BMP3 siRNA increased the proinflammatory chemokines and migration of FLS stimulated with TNF-α. Inhibition of BMP3 expression also increased expression of IL-6, IL-1β, IL-17A, CCL-2, CCL-3, VCAM-1, MMP-3, and MMP-9, but not TIMP-1, in AIA and RA FLS. Correspondingly, induction of BMP3 overexpression through intra-articular injection of ad-BMP3 diminished arthritis severity in AIA rats. We also found that BMP3 may inhibit activation of TGF-β1/Smad signaling. These data indicate that BMP3 may suppress the proliferation and migration of FLS via the TGF-β1/Smad signaling pathway.

## INTRODUCTION

Rheumatoid arthritis (RA) is an autoimmune disease characterized by synovial inflammation and hyperplasia, pannus formation, and erosion of adjacent articular cartilage and bone leading to joint dysfunction [1, 2]. Although its pathogenesis remains unclear, an increasing number of studies indicate that fibroblast-like synoviocytes (FLS) are a key component of invasive pannus and contribute to the development of RA [3, 4]. In RA, activated FLS invade the extracellular matrix and secrete proinflammatory factors [5], matrix metalloproteinases (MMPs) [6, 7], chemokines [8], and cell adhesion molecules [9], which destroy articular cartilage and bone and exacerbate joint dysfunction. Therefore, inhibition of FLS migration and the inflammatory response could be a target in the treatment of RA.

FLS secrete several cytokines, including the proinflammatory factors TNF-α, IL-6, and IL-1β [10–12]; transforming growth factor-β (TGF-β) [13, 14]; chemokines [8]; and cell adhesion molecules [15]. TGF-β/Smad not only regulates several physiologic processes of bone development, but also is involved in inflammation and fibrosis of FLS in RA [14, 16]. Bone morphogenetic proteins (BMPs), as members of the TGF-β superfamily, are secreted signaling proteins [17]. They also contribute to the development of cartilage and bone [18]. Accumulating evidence indicates that BMPs participate in regulating the inflammatory response, migration, and invasion of FLS [14, 16, 19].

BMP3, which is an antagonist of osteogenic BMPs [20, 21], inhibits the proliferation of colorectal cancer cells [22]. Similarly, forced expression of BMP3 decreases cell proliferation and viability and increases apoptosis of biliary cancer cells [23]. More importantly, expression of BMP3 mRNA has been found to be decreased in arthritic synovium compared with nonarthritic synovium [24].

To further elucidate the relationship between BMP3 and proinflammatory cytokines and chemokines and migration of FLS in RA, we studied the effect of BMP3 expression on the formation of proinflammatory cytokines and chemokines and the migration of FLS.

## RESULTS

### BMP3 expression is significantly downregulated in RA

**Figure 1:**
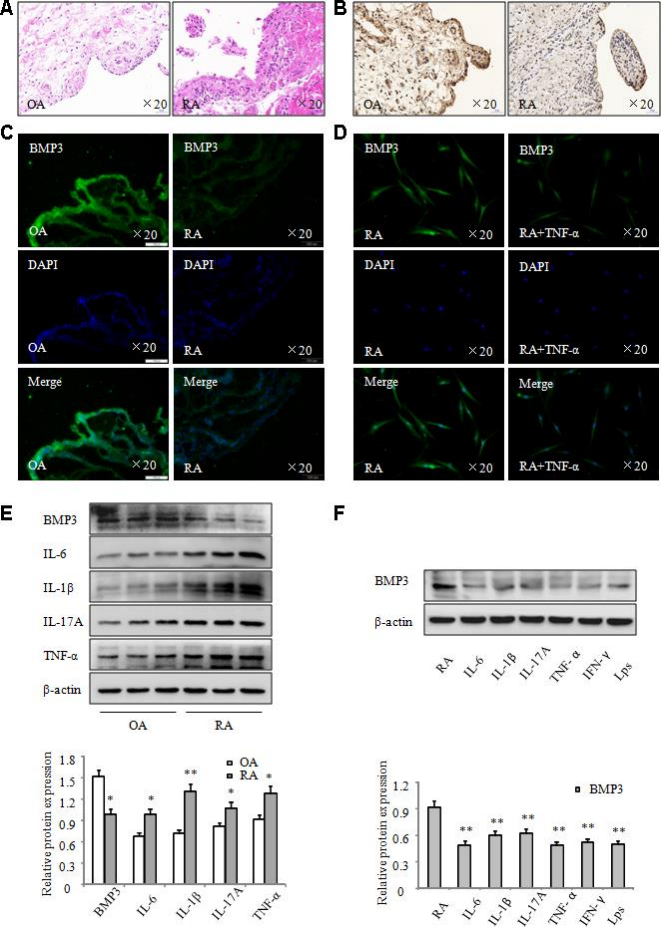
**BMP3 expression was significantly downregulated in RA.** (**A**) Representative H&E staining of OA and RA synovial tissues (original magnification, ×20). (**B**) BMP3 expression in OA and RA synovial tissues was analyzed using IHC staining (original magnification, ×20). (**C**) BMP3 expression in OA and RA synovial tissues was analyzed using immunofluorescence staining (original magnification, ×20). (**D**) BMP3 expression in RA FLS treated with TNF-α was analyzed using immunofluorescence staining (original magnification, ×10). (**E**) The protein levels of BMP3 in OA and RA synovial tissues were analyzed using western blot. (**F**) The protein levels of BMP3 in RA FLS treated with various inflammation factors were analyzed using western blot. All values are expressed as the mean ± SD. ^*^P < 0.05, ^**^P < 0.01 vs control group.

To explore the role of BMP3 in RA, we collected synovial tissues from patients with osteoarthritis (OA) and RA. We then performed histopathologic analysis (Figure 1A), and the results indicated that inflammatory cell infiltration and synovial hyperplasia were significantly increased in RA synovial tissues. In addition, immunohistochemical analysis (Figure 1B) demonstrated that BMP3 expression was significantly downregulated in RA synovial tissues compared to expression in OA synovial tissues. Immunofluorescence staining also verified that BMP3 expression was notably decreased in RA synovial tissues (Figure 1C) and in RA FLS treated with TNF-α (Figure 1D). Western blot results also demonstrated that BMP3 expression was significantly downregulated in RA synovial tissues (Figure 1E). Conversely, expression of IL-6, IL-1β, IL-17A, and TNF-α was significantly increased in RA synovial tissues. Importantly, after RA FLS were treated with various inflammatory factors in vitro, such as IL-6, IL-1β, IL-17A, TNF-α, IFN-γ, and LPS, western blot results showed that BMP3 expression was decreased to different degrees (Figure 1F). Thus, these results indicate that BMP3 expression is significantly decreased in RA synovial tissues and FLS.

### BMP3 expression is significantly downregulated in AIA

**Figure 2:**
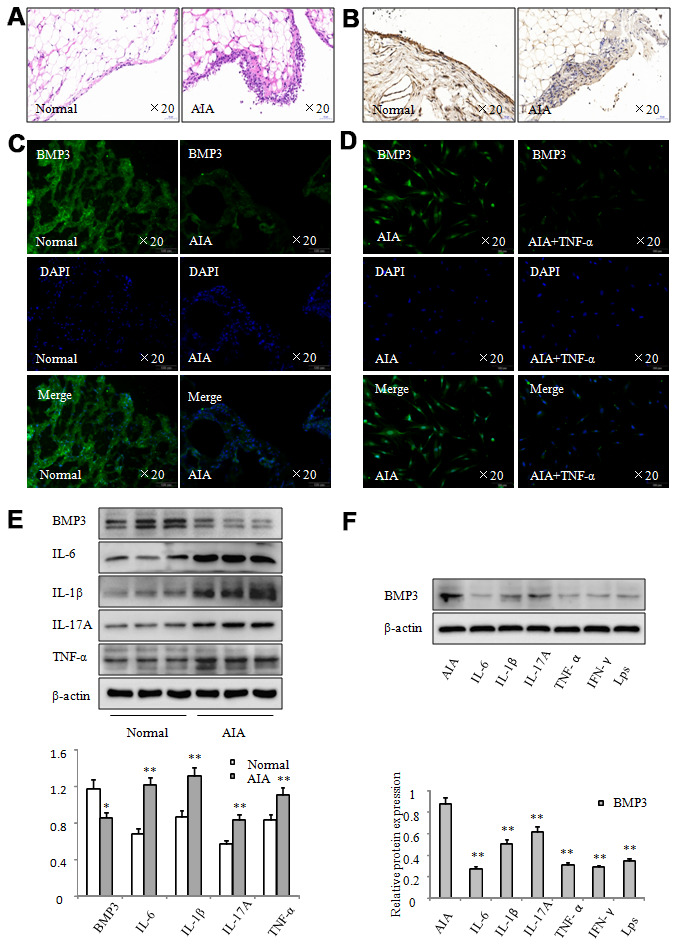
**BMP3 expression was significantly downregulated in AIA.** (**A**) Representative H&E staining of normal and AIA rat synovial tissues (original magnification, ×20). (**B**) BMP3 expression in normal and AIA rat synovial tissues was analyzed using IHC staining (original magnification, ×20). (**C**) BMP3 expression in normal and AIA rat synovial tissues was analyzed using immunofluorescence staining (original magnification, ×20). (**D**) BMP3 expression in FLS from AIA rats treated with TNF-α was analyzed using immunofluorescence staining (original magnification, ×10). (**E**) BMP3 protein levels in normal and AIA synovial tissues were analyzed using western blot. (**F**) BMP3 protein levels in AIA FLS treated with various inflammation factors were analyzed using western blot. All values are expressed as the mean ± SD. ^*^P < 0.05, ^**^P < 0.01 vs control group.

To further investigate the role of BMP3 in RA, we also created an AIA model using injection with the complete Freund’s adjuvant. Histopathologic analysis (Figure 2A) confirmed that the AIA model was successfully established. Our results showed that inflammatory cell infiltration and synovial hyperplasia were significantly increased in the synovial tissue of AIA rats. Furthermore, immunohistochemical analysis (Figure 2B) showed that BMP3 expression was significantly downregulated in AIA synovial tissue. We also measured BMP3 protein expression in synovial tissues and FLS using immunofluorescence staining (Figure 2C and 2D). Our results showed that BMP3 expression was decreased in the AIA synovial tissues compared with that in normal synovial tissues (Figure 2C). In addition, BMP3 expression was lower in AIA FLS treated with TNF-α (Figure 2D). Western blot analysis also demonstrated that BMP3 expression was significantly downregulated in AIA rat synovial tissues (Figure 2E). However, the expression of IL-6, IL-1β, IL-17A, and TNF-α was concomitantly increased in AIA synovial tissues. More significantly, after the AIA FLS were treated with various inflammation factors in vitro, such as IL-6, IL-1β, IL-17A, TNF-α, IFN-γ, and LPS, western blot results demonstrated that BMP3 expression was decreased to different degrees (Figure 2F). Thus, these results indicate that BMP3 expression is reduced in AIA synovial tissues and FLS.

### Inhibition of BMP3 expression increases proinflammatory cytokines and chemokines in RA and AIA FLS

**Figure 3:**
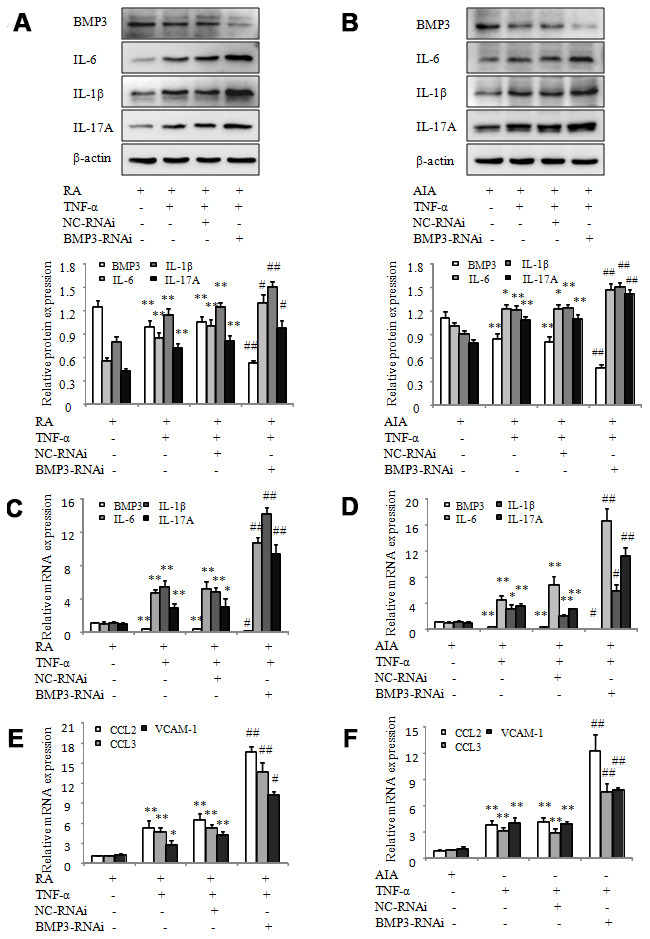
**BMP3 siRNA silencing increases the proinflammatory cytokines and chemokines in RA and AIA FLS.** (**A**) The protein levels of BMP3, IL-6, IL-1β, and IL-17A in TNF-α–treated RA FLS transfected with BMP3 siRNA were analyzed using western blot. (**B**) The protein levels of BMP3, IL-6, IL-1β, and IL-17A in TNF-α–treated AIA FLS transfected with BMP3 siRNA were analyzed using western blot. (**C**) The mRNA levels of BMP3, IL-6, IL-1β, and IL-17A in TNF-α–treated RA FLS transfected with BMP3 siRNA were analyzed using qPCR. (**D**) The mRNA levels of BMP3, IL-6, IL-1β, and IL-17A in TNF-α–treated AIA FLS transfected with BMP3 siRNA were analyzed using qPCR. (**E**) The mRNA levels of CCL2, CCL3, and VCAM-1 in TNF-α–treated RA FLS transfected with BMP3 siRNA were analyzed using qPCR. (**F**) The mRNA levels of CCL2, CCL3, and VCAM-1 in TNF-α–treated AIA FLS transfected with BMP3 siRNA were analyzed using qPCR. All values are expressed as the mean ± SD. ^*^P < 0.05, ^**^P < 0.01 vs AIA group. ^#^*P* < 0.05, ^##^*P* < 0.01 vs NC-RNAi group.

To investigate the significance of BMP3 in RA FLS, specific siRNA for BMP3 was used to knock down gene expression in TNF-α–treated RA FLS. As shown in Figure 3A and 3C, western blot and quantitative polymerase chain reaction (qPCR) results showed that BMP3 expression was significantly reduced in TNF-α–treated RA FLS transfected with BMP3-RNAi compared to the cells transfected with NC-RNAi. In addition, the results of Western blot and qPCR also showed that BMP3 expression was notably reduced in TNF-α–treated AIA FLS transfected with BMP3-RNAi (Figure 3B and 3D). More significantly, after treatment with TNF-α, the results of western blot and qPCR indicated that expression of the proinflammatory cytokines IL-6, IL-1β, and IL-17A was markedly upregulated by BMP3-RNAi in RA FLS (Figure 3A and 3C). As expected, the results of western blot and qPCR indicated that the expression of the proinflammatory cytokines IL-6, IL-1β, and IL-17A was also markedly upregulated by BMP3-RNAi in TNF-α–treated AIA FLS (Figure 3B and 3D). Moreover, qPCR results showed that CCL-2, CCL-3, and VCAM-1 mRNA expression was upregulated in TNF-α–treated RA FLS after transfection with BMP3-RNAi (Figure 3E). mRNA expression of CCL-2, CCL-3, and VCAM-1 was also significantly upregulated in TNF-α–treated AIA FLS after transfection with BMP3-RNAi (Figure 3F). Thus, these results demonstrate that inhibition of BMP3 expression increases proinflammatory cytokines and chemokines in RA and AIA FLS.

### Inhibition of BMP3 expression promotes the migration of RA and AIA FLS

**Figure 4:**
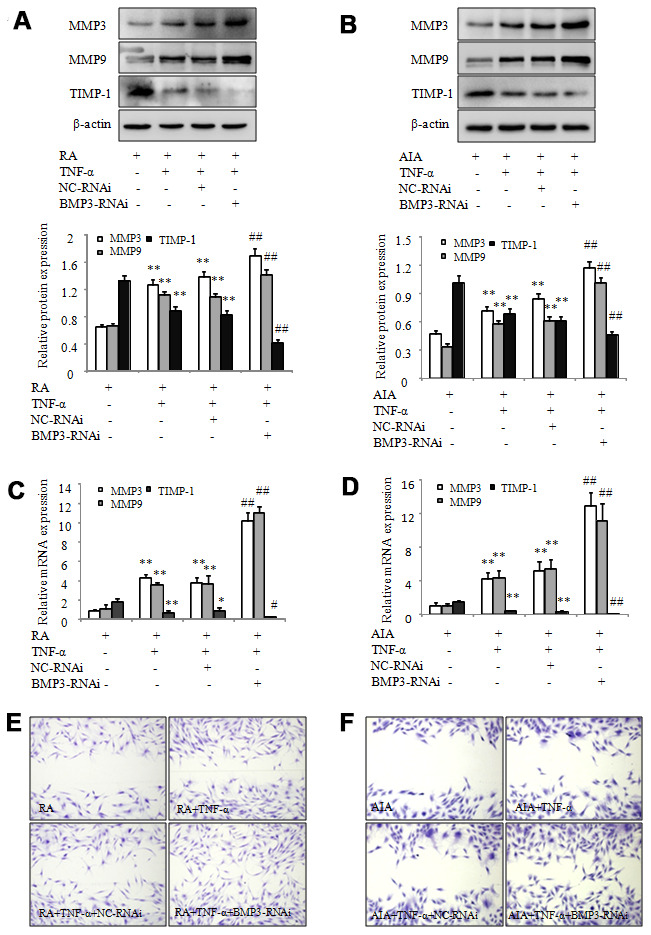
**BMP3 siRNA silencing promotes the migration of RA and AIA FLS.** (**A**) The protein levels of MMP-3, MMP-9, and TIMP-1 in TNF-α–treated RA FLS transfected with BMP3 siRNA were analyzed using western blot. (**B**) The protein levels of MMP-3, MMP-9, and TIMP-1 in TNF-α–treated AIA FLS transfected with BMP3 siRNA were analyzed using western blot. (**C**) The mRNA levels of MMP-3, MMP-9, and TIMP-1 in TNF-α–treated RA FLS transfected with BMP3 siRNA were analyzed using qPCR. (**D**) The mRNA levels of MMP-3, MMP-9, and TIMP-1 in TNF-α–treated AIA FLS transfected with BMP3 siRNA were analyzed using qPCR. (**E**) TNF-α–treated AIA FLS were transfected with BMP3 siRNA, and their migration into the wound-healing site after 24 hours was photographed (original magnification, ×10). (**F**) TNF-α–treated AIA FLS were transfected with BMP3 siRNA, and their migration into the wound-healing site after 24 hours was photographed (original magnification, ×10). All values are expressed as the mean ± SD. ^*^P < 0.05, ^**^P < 0.01 vs RA group. ^#^*P* < 0.05, ^##^*P* < 0.01 vs NC-RNAi group.

Based on the previous results, we explored the effect of BMP3 on the migration of RA and AIA FLS after transfection with specific siRNA for BMP3. As shown in Figure 4A and 4C, the results of western blot and qPCR demonstrated that the protein and mRNA levels of MMP-3 and MMP-9 were higher in TNF-α–treated RA FLS transfected with BMP3-RNAi compared to the cells transfected with NC-RNAi. Conversely, the protein and mRNA levels of TIMP-1 were downregulated in RA FLS treated with TNF-α after transfection with BMP3-RNAi (Figure 4A and 4C). In addition, the results of Western blot and qPCR also showed that MMP-3 and MMP-9 expression was significantly upregulated in TNF-α–treated AIA FLS transfected with BMP3-RNAi (Figure 4B and 4D), whereas the protein and mRNA expression of TIMP-1 was downregulated in AIA FLS under the same conditions (Figure 4B and 4D). We also performed a wound-healing assay, and the results showed that the migration ability of RA FLS was significantly increased after transfection with BMP3-RNAi (Figure 4E). The migration of AIA FLS was also increased after BMP3-RNAi transfection (Figure 4F). Therefore, our findings suggest that inhibition of BMP3 expression promotes the migration of RA and AIA FLS and contributes to the progression of RA.

### Overexpression of BMP3 decreases proinflammatory cytokines and chemokines in RA and AIA FLS

**Figure 5:**
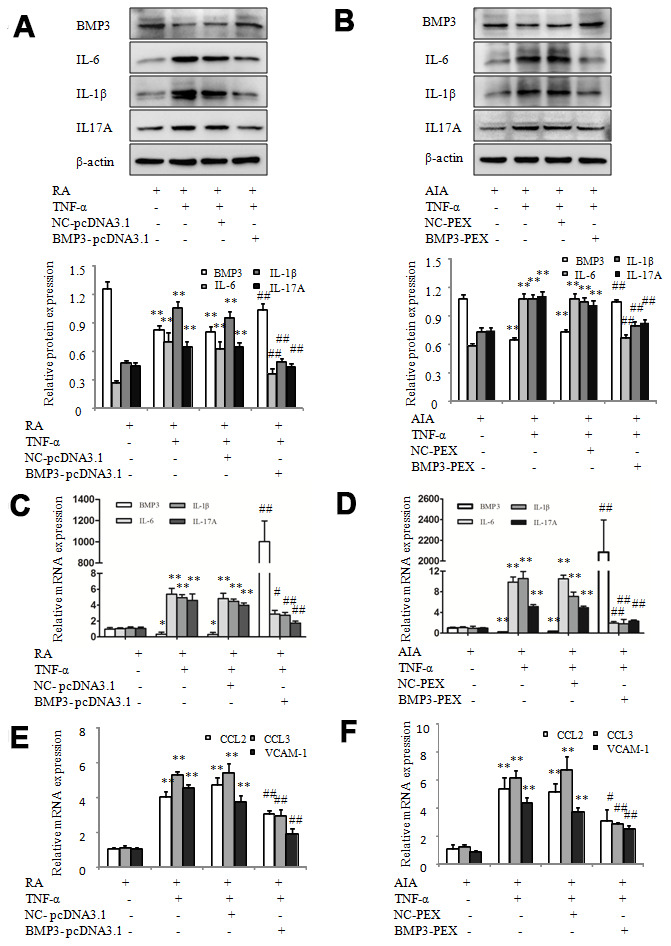
**Overexpression of BMP3 decreases proinflammatory cytokines and chemokines in RA and AIA FLS.** (**A**) The protein levels of BMP3, IL-6, IL-1β, and IL-17A in TNF-α–treated RA FLS transfected with BMP3-pcDNA3.1 were analyzed using western blot. (**B**) The protein levels of BMP3, IL-6, IL-1β, and IL-17A in TNF-α–treated AIA FLS transfected with BMP3-PEX were analyzed using western blot. (**C**) The mRNA levels of BMP3, IL-6, IL-1β, and IL-17A in TNF-α–treated RA FLS transfected with BMP3-pcDNA3.1 were analyzed using qPCR. (**D**) The mRNA levels of BMP3, IL-6, IL-1β, and IL-17A in TNF-α–treated AIA FLS transfected with BMP3-PEX were analyzed using qPCR. (**E**) The mRNA levels of CCL2, CCL3, and VCAM-1 in TNF-α–treated RA FLS transfected with BMP3-pcDNA3.1 were analyzed using qPCR. (**F**) The mRNA levels of CCL2, CCL3, and VCAM-1 in TNF-α–treated AIA FLS transfected with BMP3-PEX were analyzed using qPCR. All values are expressed as the mean ± SD. ^*^P < 0.05, ^**^P < 0.01 vs AIA group. ^#^*P* < 0.05, ^##^*P* < 0.01 vs NC-PEX group.

To further study the effect of BMP3 on the proinflammatory cytokines and chemokines of RA FLS, we used the overexpression plasmids BMP3-pcDNA3.1 for humans and BMP3-PEX for rats to induce overexpression of BMP3 in TNF-α–treated RA and AIA FLS, respectively. As shown in Figure 5A and 5C, the western blot and qPCR results showed that BMP3 expression was higher in TNF-α–treated RA FLS transfected with BMP3-pcDNA3.1, compared to the cells transfected with NC-pcDNA3.1 (the negative control). Furthermore, the protein and mRNA levels of the proinflammatory cytokines IL-6, IL-1β, and IL-17A were significantly downregulated by BMP3-pcDNA3.1 in RA FLS treated with TNF-α (Figure 5A and 5C). In addition, the western blot and qPCR results showed that the expression of BMP3 was significantly higher in TNF-α–treated AIA FLS transfected with BMP3-PEX, compared to the cells transfected with empty vector (NC-PEX) (Figure 5B and 5D). The protein and mRNA levels of the proinflammatory cytokines IL-6, IL-1β, and IL-17A were significantly decreased by BMP3-PEX in AIA FLS treated with TNF-α (Figure 5B and 5D). More significantly, as shown in Figure 5E, after transfection with BMP3-pcDNA3.1, the qPCR results showed that the chemokines CCL-2, CCL-3, and VCAM-1 were notably reduced in TNF-α–treated RA FLS. Also, the mRNA expression of the chemokines CCL-2, CCL-3, and VCAM-1 was significantly reduced after transfection with BMP3-PEX in TNF-α–treated AIA FLS (Figure 5F). Thus, these results demonstrate that BMP3 overexpression decreases the levels of proinflammatory cytokines and chemokines in RA.

### Overexpression of BMP3 inhibits the migration of RA and AIA FLS

**Figure 6:**
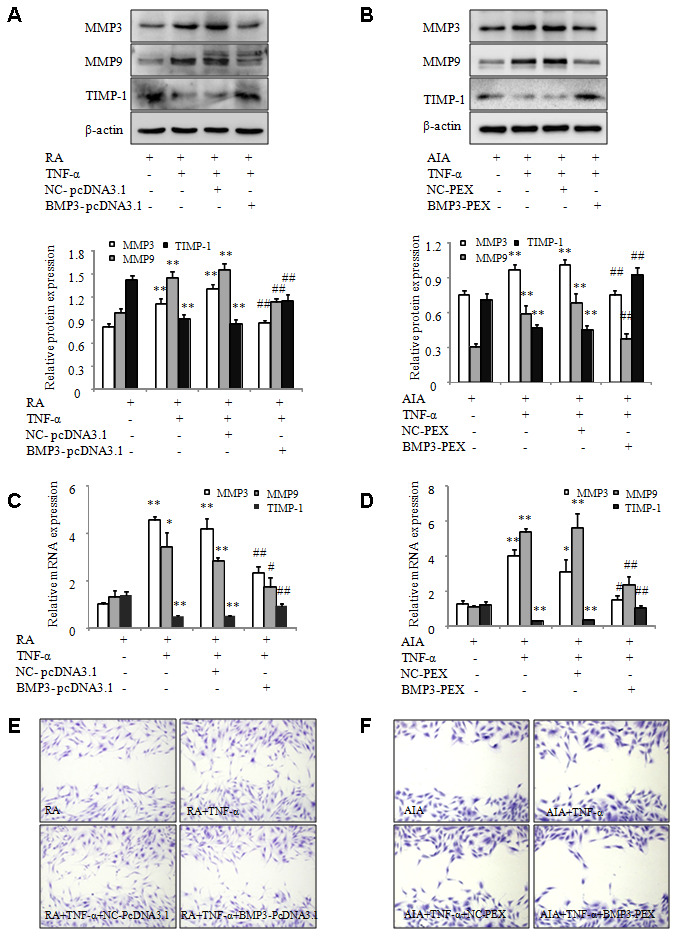
**Overexpression of BMP3 inhibits the migration of RA and AIA FLS.** (**A**) The protein levels of MMP-3, MMP-9, and TIMP-1 in TNF-α–treated RA FLS transfected with BMP3-pcDNA3.1 were analyzed using western blot. (**B**) The protein levels of MMP-3, MMP-9, and TIMP-1 in TNF-α–treated AIA FLS transfected with BMP3-PEX were analyzed using western blot. (**C**) The mRNA levels of MMP-3, MMP-9, and TIMP-1 in TNF-α–treated RA FLS transfected with BMP3-pcDNA3.1 were analyzed using qPCR. (**D**) The mRNA levels of MMP-3, MMP-9, and TIMP-1 in TNF-α–treated AIA FLS transfected with BMP3-PEX were analyzed using qPCR. (**E**) TNF-α–treated RA FLS were transfected with BMP3-pcDNA3.1, and their migration into the wound-healing site after 24 hours was photographed (original magnification, ×10). (**F**) TNF-α–treated AIA FLS were transfected with BMP3-PEX, and their migration into the wound-healing site after 24 hours was photographed (original magnification, ×10). All values are expressed as the mean ± SD. ^*^P < 0.05, ^**^P < 0.01 vs RA group. ^#^*P* < 0.05, ^##^*P* < 0.01 vs NC-pcDNA3.1 group.

We measured the effect of BMP3 on the migration of RA and AIA FLS after transfection with BMP3 overexpression plasmids. As expected, the western blot and qPCR results showed that MMP-3 and MMP-9 expression was significantly upregulated in RA FLS stimulated by TNF-α. However, compared with the cells transfected with NC-pcDNA3.1, the increases in MMP-3 and MMP-9 were significantly inhibited by BMP3-pcDNA3.1 in TNF-α–treated RA FLS (Figure 6A and 6C). Conversely, the expression of TIMP-1 was significantly increased in RA FLS treated with TNF-α after transfection with BMP3-pcDNA3.1 (Figure 6A and 6C). In TNF-α–treated AIA FLS transfected with BMP3-PEX, the protein and mRNA levels of MMP-3 and MMP-9 were significantly downregulated (Figure 6B and 6D), whereas the expression of TIMP-1 was significantly upregulated (Figure 6B and 6D). In addition, the wound-healing assay indicated that the migration of RA FLS was suppressed after overexpression of BMP3 (Figure 6E). The migration of AIA FLS was also inhibited by BMP3-PEX transfection, compared to the negative control (Figure 6F). Taken together, these data demonstrate that BMP3 overexpression inhibits the migration of FLS in RA and AIA and that BMP3 may play a role in the pathogenesis of RA.

### BMP3 regulates the migration and proinflammatory response of FLS and may be associated with the TGF-β1/Smad signaling pathway

**Figure 7:**
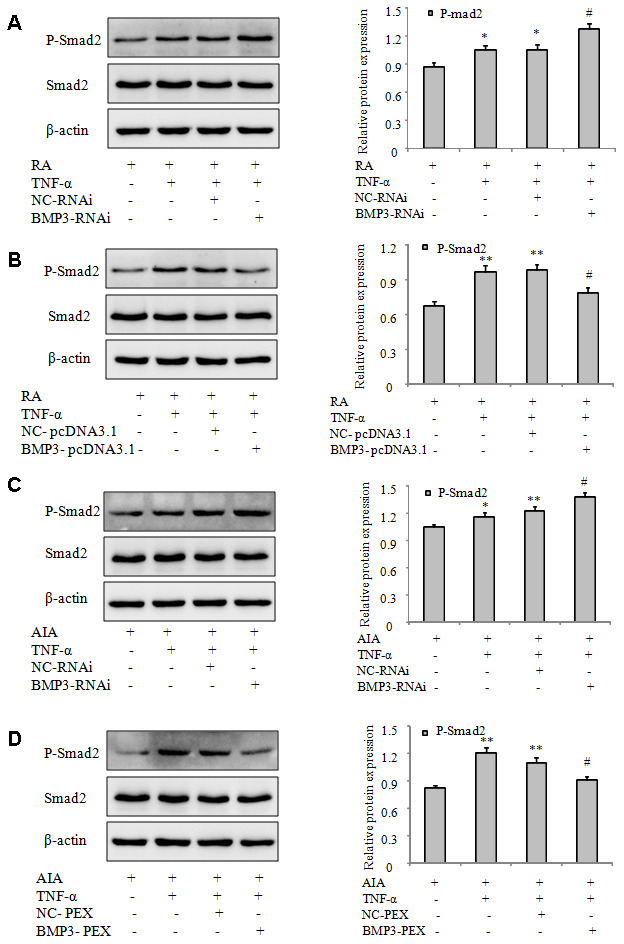
**BMP3 regulates the proinflammatory response and migration of FLS and may be associated with the TGF-β1/Smad signaling pathway.** (**A**) The protein levels of p-Smad2 in RA FLS transfected with BMP3 siRNA were analyzed using western blot. (**B**) The protein levels of p-Smad2 in RA FLS transfected with BMP3-PEX were analyzed using western blot. (**C**) The protein levels of p-Smad2 in AIA FLS transfected with BMP3-RNAi were analyzed using western blot. (**D**) The protein levels of p-Smad2 in AIA FLS transfected with BMP3-pcDNA3.1 were analyzed using western blot. All values are expressed as the mean ± SD. ^*^P < 0.05, ^**^P < 0.01 vs normal group. ^#^*P* < 0.05, ^##^*P* < 0.01 vs model group.

BMP3 is associated with the TGF-β1/Smad signaling pathway in pathophysiologic mechanisms [25]. To investigate whether BMP3 regulates proinflammatory cytokines, chemokines, and migration through the Smad signaling pathway in FLS, we measured the protein levels of phosphorylated (p)-Smad2, a TGF-β1/Smad pathway activation marker. Western blot analysis demonstrated upregulation of p-Smad2 in RA and AIA when FLS were treated with TNF-α. In particular, expression of p-Smad2 was significantly increased after BMP3 silencing (Figure 7A and 7C) but reduced after BMP3 overexpression (Figure 7B and 7D). These results indicate that BMP3 may regulate FLS migration and secretion of proinflammatory cytokines through the Smad signaling pathway.

### Induction of BMP3 overexpression by adenovirus in vivo alleviated arthritis severity in AIA rats

**Figure 8:**
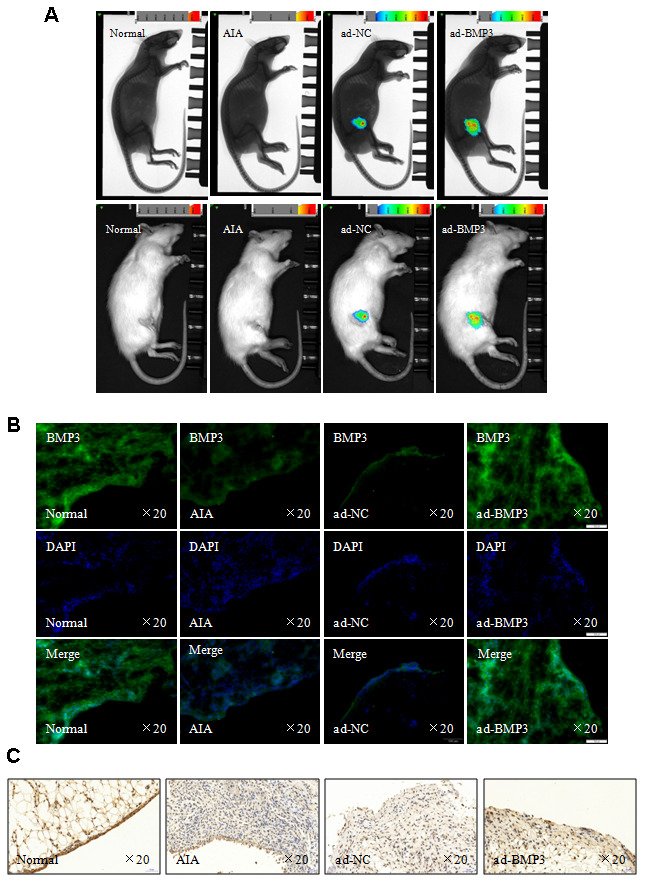
**Overexpression of BMP3 by adenovirus in vivo alleviated arthritis severity in AIA rats.** (**A**) In vivo imaging of normal and AIA rats. (**B**) Overexpression of BMP3 in AIA rat synovial tissues injected with ad-BMP3 was analyzed using immunofluorescence staining (original magnification, ×20). (**C**) BMP3 expression in AIA rat synovial tissues injected with ad-BMP3 was analyzed using IHC staining (original magnification, ×20).

**Figure 9:**
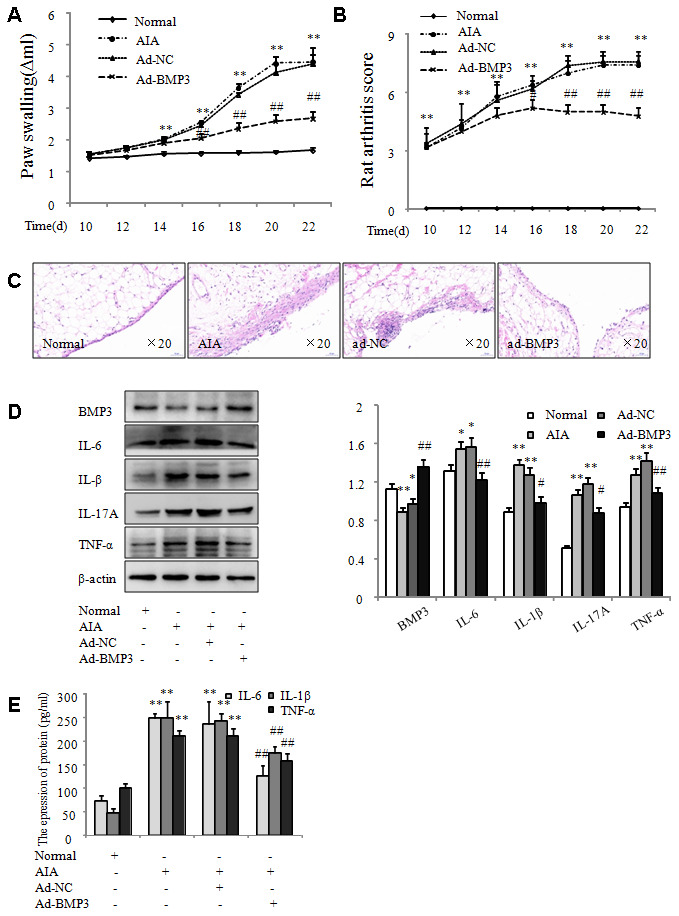
**Overexpression of BMP3 by adenovirus in vivo alleviated arthritis severity in AIA rats.** (**A**) The swelling of ankle joints was quantified using a plethysmometer. (**B**) Arthritis scores were reduced in AIA rats injected with ad-BMP3. (**C**) Representative H&E staining of normal and AIA rat synovial tissues (original magnification, ×20). (**D**) BMP3 protein levels in normal and AIA synovial tissues were analyzed using western blot. (**E**) Levels of IL-6, IL-1β, and TNF-α expression in the serum of AIA rats injected with ad-BMP3. All values are expressed as the mean ± SD. ^*^P < 0.05, ^**^P < 0.01 vs normal group. ^#^*P* < 0.05, ^##^*P* < 0.01 vs ad-NC group.

To explore the effect of BMP3 in synovial tissues of AIA in vivo, adenovirus carrying Rattus BMP3 (ad-BMP3) was injected intra-articularly into the knees of AIA rats to induce overexpression of BMP3, and adenovirus carrying LUC (ad-LUC) was used as a control. First, in vivo imaging showed that adenovirus carrying BMP3 and LUC replicated successfully in rat joints (Figure 8A). Second, using immunofluorescence staining and immunohistochemical analysis to detect BMP3 expression in synovial tissues of AIA, we found that BMP3 expression was significantly higher in the synovial tissues of ad-BMP3–injected joints compared with ad-LUC–injected joints (Figure 8B and 8C). Overexpression of BMP3 also reduced paw swelling caused by AIA (Figure 9A) and the articular index (Figure 9B). More importantly, histopathologic analysis confirmed that intra-articular injection of ad-BMP3 significantly reduced inflammatory cell infiltration and synovial hyperplasia in the synovial tissue of AIA rats (Figure 9C). In addition, the results of western blot analysis showed that the proinflammatory cytokines IL-6, IL-1β, IL-17A, and TNF-α were all significantly downregulated by intra-articular injection of ad-BMP3 in AIA synovial tissues (Figure 9D). IL-6, IL-1β, and TNF-α expression was also significantly downregulated in the serum of AIA rats injected with ad-BMP3 (Figure 9E). Thus, our results confirm that overexpression of BMP3 induced by intra-articular injection of ad-BMP3 alleviates arthritis severity in AIA rats.

## DISCUSSION

The results of this study demonstrated the following: (1) BMP3 expression is significantly downregulated in RA and AIA synovial tissues; (2) BMP3-RNAi increased the proinflammatory response and migration of RA and AIA FLS; (3) the overexpression vector of BMP3 suppressed the proinflammatory response and migration of RA and AIA FLS; (4) overexpression of BMP3 decreases the proinflammatory response and migration of FLS and BMP3 expression may be closely associated with the TGF-β1/Smad signaling pathway; and (5) induction of BMP3 overexpression by adenovirus in vivo alleviated arthritis severity in AIA rats.

The AIA rat model is widely used to study the pathogenesis of RA [26]. The AIA model has similar characteristics as RA with respect to histology and immunology and is a well-established model for evaluating treatment for RA [27]. Hence, we chose AIA to investigate the role of BMP3 in RA. RA is a systemic autoimmune disease characterized by synovial inflammation and hyperplasia and erosion of the synovial joints, leading to articular destruction and functional disability [28]. It is clear that various inflammatory cells are activated in RA, such as T cells, B cells, and FLS [29]. Accumulating evidence indicates that hyperplastic FLS potentially promote macrophage and lymphocyte infiltration, recruitment, and retention via the production of chemokines, cell adhesion molecules, proinflammatory cytokines, and extracellular matrix proteins [30–32]. Although FLS have been shown to play an important role in RA, the mechanisms underlying the activation of FLS remain unresolved.

BMP3, a member of the BMP family, has been shown to inhibit the proliferation of colorectal cancer cells [22] and biliary cancer cells [23]. BMP3 mRNA expression has also been shown to be lower in arthritic synovium than in the synovium of nonarthritic controls [24].

In this study, we explored whether BMP3 plays a role in regulating the proinflammatory response and migration of FLS. In this study, we show that BMP3 expression is significantly reduced in RA and AIA synovial tissues using western blot, qRT-PCR, immunohistochemical, and immunofluorescence analysis. Notably, after AIA and RA FLS were treated with various inflammation factors in vitro, the expression of BMP3 was decreased to different degrees. TNF-α is one of the major cytokines involved in RA. It has been shown that stimulation using 10 ng/mL of TNF-α results in the activation of RA FLS and increases production of inflammatory cytokines [10]. It has also been reported that TNF-α stimulation affects the expression levels of certain genes in RA FLS [33]. In the current study, we explored whether BMP3 affects the proinflammatory response and migration of RA and AIA FLS.

Western blot and qRT-PCR analyses revealed that inhibition of BMP3 expression using BMP3-RNAi resulted in the overexpression of the proinflammatory cytokines IL-6, IL-1β, and IL-17A in RA and AIA FLS treated with TNF-α. Studies indicate that the secretion of inflammatory factors leads to the activation and migration of RA FLS and exacerbates the inflammatory response [30, 34]. Furthermore, inhibition of BMP3 expression using BMP3-RNAi increases the expression of CCL-2, CCL-3, VCAM-1, MMP-3, and MMP-9 in RA and AIA FLS treated with TNF-α, whereas TIMP-1 expression is decreased. Macrophages and FLS in synovial tissues are a major source of chemokines [35]. Many chemokines produced by RA synovium may facilitate the recruitment and activation of FLS in synovial tissue and subsequently aggravate inflammation and bone destruction in the progression of RA [36, 37]. MMP-9 promotes the migration and invasion of FLS in collagen-induced arthritis in mice [38], whereas MMP-3 causes FLS to migrate to adjacent cartilage and induces articular cartilage degradation [39]. Significantly, our wound-healing assay results also suggest that inhibition of BMP3 expression increases the migration of RA and AIA FLS.

Our goal was to determine whether BMP3 overexpression reduces the proinflammatory response and migration of FLS. In this study, RA and AIA FLS treated with TNF-α were transfected with the overexpression vectors BMP3-PEX and BMP3-pcDNA3.1. Our results indicate that BMP3 overexpression downregulates the expression of proinflammatory cytokines, chemokines, MMP-3, and MMP-9, and upregulates TIMP-1 expression. Thus, BMP3 overexpression significantly reduces the proinflammatory response and migration of AIA FLS.

More importantly, inflammatory cells infiltration, arthritis scores, and paw swelling were significantly decreased by intra-articular injection of ad-BMP3 in AIA knees in vivo. The expression of IL-6, IL-1β, and TNF-α was also significantly downregulated in AIA serum. Thus, BMP3 overexpression may improve RA by suppressing the production of proinflammatory cytokines, chemokines, and MMPs of FLS.

BMP3 mediates cell activation by negatively regulating the TGF-β1/Smad signaling pathway [25]. The activation of p-Smad kinases plays a vital role in cell growth, proliferation, and invasion [14]. Our data also indicate that inhibition of BMP3 by BMP3-RNAi significantly increases p-Smad2 expression in AIA and RA FLS. In particular, overexpression of BMP3 suppressed Smad signaling, with a substantial decrease in p-Smad2 expression in AIA and RA FLS. Taken together, these data indicate that BMP3 mediates the proinflammatory cytokines and migration of FLS and might regulate the activation of the TGF-β1/Smad signaling pathway.

In conclusion, we found that BMP3 expression was significantly reduced in AIA and RA synovial tissues. BMP3 silencing or overexpression resulted in promotion or reduction of the proinflammatory response and migration of FLS, respectively. Our findings suggest that BMP3 plays a pivotal role in the proinflammatory response and migration of FLS through activation of the TGF-β1/Smad signaling pathway. Thus, we believe that BMP3 could be a new target for the treatment of RA.

## MATERIALS AND METHODS

### Materials and reagents

Complete Freund’s adjuvant (CFA) was obtained from Chondrex, Inc. (USA). D-luciferin was purchased from Abcam (UK). Rabbit anti-BMP3, anti-TNF-a, and anti-TIMP metallopeptidase inhibitor 1 (TIMP-1) antibodies were obtained from Abcam (UK). Rabbit anti-MMP-3 and anti-MMP-9 antibodies were acquired from Merck Millipore (USA). Rabbit anti-IL-6, anti-IL-1β, and anti-IL-17A were purchased from Bioworld (USA). The rat anti-β-actin antibody was purchased from CST (USA). A horseradish peroxidase (HRP)-labeled goat anti-rabbit immunoglobulin (IgG) was procured from Zhongshan Biotechnology Corporation (China). BMP3, MMP-3, MMP-9, TIMP-1, TNF-a, IL-6, IL-1β, IL-17A, CCL-2, CCL-3, VCAM-1, and β-actin primers were synthesized by the Shanghai Sangon Biological and Technological Company (China).

### Human synovial tissue

Human synovial tissue was obtained from patients with RA (n = 5) according to the RA criteria during joint synovectomies revised in 1987 by the American College of Rheumatology. Osteoarthritis (OA, n = 5) patients served as controls. All patients signed informed consent forms to participate in the study. The ethics board of Anhui Medical University approved the study protocol, and synovial tissue specimens were collected according to organizational guidelines. Written informed consent was obtained from all subjects.

### Adjuvant-induced arthritis (AIA) model

All Sprague-Dawley (SD) rats (120–160 g, female) were purchased from the Laboratory Animal Center of Anhui Medical University. The animals’ experimental protocols were approved by the Animal Care and Use Committee of Anhui Medical University. Adult SD rats were intradermally injected with 0.1 mL of CFA per 100 g of body weight in the left paw to induce AIA. At the same time, control group rats were treated with normal saline on day 0. The right knees of AIA rats were intra-articularly injected with 0.1 mL of adenovirus carrying Rattus BMP3 (ad-BMP3) or ad-LUC on day 8. All rats were killed on day 23 after adjuvant injection.

### Histopathology

Rat and human synovium specimens were fixed with 4% paraformaldehyde for 24 hours and then embedded in paraffin. Immunohistochemistry analysis and hematoxylin and eosin (H&E) staining were performed according to a standard procedure. The Olympus BX-51 microscope was used to photograph pathologic changes.

### ELISA assay

Rats were killed at 23 days, and serum was collected through the abdominal aorta. The levels of IL-6, IL-1β, and TNF-α in serum or FLS supernatant were analyzed using ELISA Kit (Wuhan ColorfulGene Biological Technology Co., Ltd., China) according to the manufacturer’s procedure.

### Cell culture and treatment

FLS were derived from the synovial tissues of the RA patients and AIA rats. The cells were cultured in cell culture flasks in high-glucose DMEM medium (Hyclone, USA) containing 20% (v/v) fetal bovine serum (FBS) (Gibco, USA), 100 mg/mL of streptomycin, and 100 U/mL of penicillin (both from Beyotime, China). All cells were cultured at 37°C at an atmosphere of 5% CO_2_. The FLS of 3-8 generation were used in the subsequent experiments.

We explored the effects of various inflammatory treatments on the expression of BMP3 in FLS. First, we seeded FLS into 6-well plate for 24 hours. Subsequently, the FLS were incubated with IL-6 (5 ng/mL), IL-1β (2 ng/mL), IL-17A (10 ng/mL), TNF-α (10 ng/mL), IFN-γ (10 ng/mL), and LPS (1 μg/mL) for 48 hours, respectively. Then the FLS were collected and analyzed using qPCR and Western blot.

### Immunofluorescence staining

FLS were cultured in DMEM with 20% FBS at a density of 1–2 × 10^5^ cells/mL. FLS were stimulated with TNF-α for 48 hours and then fixed with methanol. Immunofluorescence staining was performed with rabbit anti-BMP3 (Abcam, UK) and anti-Vimentin (Cell Signaling, USA). Alexa Fluor 488–conjugated goat anti-rabbit IgG (Zhongshan Biotechnology Corporation, China) was used as the secondary antibody. Counterstaining of nuclei was performed with 4′,6-diamidino-2-phenylindole (DAPI; Beyotime, China). Pathologic changes were observed and photographed using an Olympus BX-51 microscope.

### Small interfering RNA transfection

Small interfering RNA targeting BMP3 (BMP3-RNAi) was purchased from GenePharma Corporation (China). The BMP3-RNAi (rat) sense strand is 5′-CCAAAGUCUUUGAAGCCAUTT-3′, and antisense strand is 5′-AUGGCUUCAAAGACUUUGGTT-3′. The BMP3-RNAi (human) sense strand is 5′-GCAAGACAAGGUCUCUGAAdTdT-3′, and antisense strand is 5′-UUCAGAGACCUUGUCUUGCdTdT-3′. The cultured FLS were transfected with 100 nM of small interfering RNA (RNAi) using Lipofectamine 2000 (Invitrogen, USA), according to the manufacturer’s instructions. Negative scrambled RNAi was used as control in the experiment. After transfection for 4–6 hours, we replaced the Opti-MEM with DMEM containing 20% FBS and co-cultured with TNF-α (10 ng/mL) for 48 hours, and then transfected FLS were collected for the subsequent experiments.

### Plasmid construction and transfection

Overexpression plasmid for rat BMP3-PEX was purchased from GenePharma Corporation (Shanghai, China), and human BMP3-pcDNA3.1 was purchased from Hanbio (Shanghai, China). The FLS were transfected with BMP3-PEX and BMP3-pcDNA3.1 to induce the ectopic expression of BMP3 and with the empty PEX vector (PEX) or pcDNA3.1 as a control. The transfection experiments were performed using Lipofectamine 2000 (Invitrogen, USA) according to the manufacturer’s instructions. After incubation of transfected FLS with TNF-α (10 ng/mL) for 48 hours, cells were collected for subsequent tests, such as qRT-PCR and Western blot analyses.

### Ad-BMP3 overexpression

BMP3 adenovirus (ad-BMP3) and the negative control adenovirus (ad-LUC) were purchased from Hanbio (Shanghai, China). The stock solutions of ad-BMP3 and ad-LUC contained 1 × 10^10^ plaque formation units (PFU)/mL, respectively.

### In vivo imaging

D-luciferin was dissolved in DMSO and finally formulated into a 50 mmol/L solution. The rats were anesthetized with 2% isoflurane, and 50 μL of D-luciferin solution was injected intra-articularly into the right knee of each rat. After 5–10 minutes, the rats were placed right side up in the imaging chamber. The images were captured after a continuous exposure time of 15 seconds in brightfield or x-ray using SI Imaging Amix (SI Imaging Services Co., Ltd, USA).

### Wound-healing assay

FLS were cultured in 6-well plates (1.0 × 10^6^ cells/well) in DMEM containing 20% FBS for 24 h. Subsequently, cells were scratched with a pipette tip, serum deprived, and treated with BMP3-RNAi or BMP3-PEX/BMP3-pcDNA3.1 for 24 hours. Then, the cells were fixed with methanol, stained with crystal violet, and photographed using an Olympus BX-51 microscope.

### Real-time quantitative PCR

Total RNA was extracted from FLS using TRIzol reagent (Invitrogen) according to the manufacturer’s manuals/protocol. Then, cDNA was reverse transcribed using a TAKARA kit system (Japan). The reaction was performed according to the manufacturer’s protocol using TB Green qPCR Master Mix (TAKARA, Japan). Furthermore, the mRNA expression of the genes was detected in a Pikoreal 96 real-time PCR system (Thermo Scientific, USA). All the primers we used are shown in Tables 1 and 2. The relative gene mRNA expression was analyzed using the 2−ΔΔCt method and normalized to β-actin mRNA expression.

**Table 1:** Primers sequences used for quantitative real-time PCR (Rat)

**Gene**	**Primer sequence**
BMP3	forward: 5′- CCTACCCTACCTAGCTACGTAT -3′
	reverse: 5′- GCTGGTGACATTGTTACTCATG -3′
IL-6	forward: 5′- GAGCCCACCAGGAACGAAAGTC -3′
	reverse: 5′-TGTTGTGGGTGGTATCCTCTGTGAA-3′
IL-1β	forward: 5′- TGACCCATGTGAGCTGAAAG -3′
	reverse: 5′- AGGGATTTTGTCGTTGCTTG - 3′
IL-17A	forward: 5′-TGCCTGATGCTGTTGCTGCTAC-3′
	reverse: 5′-GGTGAAGTGGAACGGTTGAGGTAG-3′
TNF-α	forward: 5′-ACTCCCAGAAAAGCAAGCAA-3′
	reverse: 5′-CAGTTCCACATCTCGGATCA-3′
CCL-2	forward: 5′-TAGCATCCACGTGCTGTCTC-3′
	reverse: 5′-TGCTGCTGGTGATTCTCTTG-3′
CCL-3	forward: 5′-ACTGCCTGCTGCTTCTCCTA-3′
	reverse: 5′-CGGTTTCTCTTGGTCAGGAA-3′
VCAM-1	forward: 5′-GTCAGCGAAGGAAACTGGAG-3′
	reverse: 5′-ACCGTGCAGTTGACAGTGAC-3′
MMP- 3	forward: 5′- ATGATGAACGATGGACAGATGA - 3′
	reverse: 5′- CATTGGCTGAGTGAAAGAGACC -3′
MMP-9	forward: 5′-CACTGTAACTGGGGGCAACT-3′
	reverse: 5′-CACTTCTTGTCAGCGTCGAA-3′
TIMP-1	forward: 5′-CATCTCTGGCC TCTGGCATC-3′
	reverse: 5′-CATAACGCTGGTATAAGGTGGTCTC-3′
β-actin	forward: 5′-TTCGCCATGGATGACGATATC-3′
	reverse: 5′-TAGGAGTCCTTCTGACCCATAC-3′

**Table 2:** Primers sequences used for quantitative real-time PCR (Human).

**Gene**	**Primer sequence**
BMP3	forward: 5′-GCTCTACTGGGGTCTTGCTG -3′
	reverse: 5′-CTGAGCCTGAAGGGTCTTGT -3′
IL-6	forward: 5′- CACACAGACAGCCACTCACC -3′
	reverse: 5′- AGTGCCTCTTTGCTGCTTTC -3′
IL-1β	forward: 5′- GGACAAGCTGAGGAAGATGC -3′
	reverse: 5′- TCGTTATCCCATGTGTCGAA - 3′
IL-17A	forward: 5′- CGGACTGTGATGGTCAACCTGAAC -3′
	reverse: 5′- GGTCCTCATTGCGGTGGAGATTC -3′
TNF-α	forward: 5′-AACCTCCTCTCTGCCATCAA -3′
	reverse: 5′-CTGAGTAGGTCACCCTTCTC -3′
CCL-2	forward: 5′- CCTTCATTCCCCAAGGGCTC -3′
	reverse: 5′- CTTCTTTGGGACACTTGCTGC -3′
CCL-3	forward: 5′- TGCAACCAGTTCTCTGCATC -3′
	reverse: 5′- TGGCTGCTCGTCTCAAAGTA -3′
VCAM-1	forward: 5′-GTCAGCGAAGGAAACTGGAG -3′
	reverse: 5′-ACCGTGCAGTTGACAGTGAC -3′
MMP- 3	forward: 5′- GGCCAGGGATTAATGGAGAT - 3′
	reverse: 5′- TGAAAGAGACCCAGGGAGTG -3′
MMP-9	forward: 5′- GTACCACGGCCAACTACGAC -3′
	reverse: 5′- GCCTTGGAAGATGAATGGAA -3′
TIMP-1	forward: 5′- TGACATCCGGTTCGTCTACA -3′
	reverse: 5′- TGATGTGCAAGAGTCCATCC -3′
β-actin	forward: 5′- GCCAACACAGTGCTGTCTGG -3′
	reverse: 5′- CTCAGGAGGAGCAATGATCTTG -3′

### Western blotting

Cultured FLS in vitro and synovial tissues were lysed using the radioimmunoprecipitation assay (RIPA) reagent containing 1% phenylmethanesulfonyl fluoride (PMSF) (Beyotime, China). The whole-cell extracts (20 mg protein) were separated by 10% sodium dodecyl sulfate–polyacrylamide gel electrophoresis (SDS-PAGE) and blotted onto PVDF membranes (Millipore, USA). After blocking with 5% milk for 2 hours, the membranes were incubated with the appropriate antibodies, which were diluted using an antibody dilution buffer (Beyotime, China). The primary antibodies against BMP3 (1:500), IL-6 (1:500), IL-1β (1:500), IL-17 (1:500), TNF-α (1:500), MMP-3 (1:500), MMP-9 (1:500), TIMP-1 (1:500), TGF-β1 (1:500), and β-actin (1:1000) were used. After incubation with the primary antibodies for 12 hours, the blot was washed three times using TBS/Tween-20 (TBST). The blot was then incubated with goat anti-mouse or rabbit HRP-conjugated antibodies at 1:5000 dilutions in TBST containing 5% skim milk for 2 hours. After washing three times with TBST, the protein bands in the western blot were analyzed using Immobilon Western Chemiluminescent HRP Substrate (Millipore).

### Statistical analysis

All data are presented as the means ± standard deviations (SDs) and were analyzed by SPSS 16.0 software. The results were from at least three experiments. The Student-Newman-Keuls test or one-way analysis of variance (ANOVA) was used to compare the means of different values. *P* values < 0.05 were considered statistically significant.
